# Correlation of Immunomodulatory Cytokines with Tumor Volume and Cerebrospinal Fluid in Vestibular Schwannoma Patients

**DOI:** 10.3390/cancers16173002

**Published:** 2024-08-29

**Authors:** Anna-Louisa Becker, Leila Scholle, Clara Helene Klause, Martin Sebastian Staege, Christian Strauss, Markus Otto, Stefan Rampp, Christian Scheller, Sandra Leisz

**Affiliations:** 1 Department of Neurosurgery, Medical Faculty, Martin Luther University Halle-Wittenberg, Ernst-Grube-Str. 40, 06120 Halle (Saale), Germany; 2 Department of Neurology, Medical Faculty, Martin Luther University Halle-Wittenberg, Ernst-Grube-Str. 40, 06120 Halle (Saale), Germany; 3 Department of Surgical and Conservative Pediatrics and Adolescent Medicine, Medical Faculty, Martin Luther University Halle-Wittenberg, Ernst-Grube-Str. 40, 06120 Halle (Saale), Germany; 4 Department of Neurosurgery, University Hospital Erlangen, Schwabachanlage 6, 91054 Erlangen, Germany; 5 Department of Neuroradiology, University Hospital Erlangen, Schwabachanlage 6, 91054 Erlangen, Germany

**Keywords:** vestibular schwannoma, acoustic neuroma, cytokines, tumor-associated macrophages, transforming growth factor, CC-chemokine ligand, growth differentiation factor

## Abstract

**Simple Summary:**

The mechanisms underlying the size progression of vestibular schwannomas remain poorly understood. Accordingly, this study was focused on investigating the potential role of messenger substances in regulating the growth of these tumors. These cytokines are produced by the tumor cells themselves or by other cells, such as immune cells. The influence of the cytokines on the size progression of tumors could facilitate the development and establishment of drug therapies for vestibular schwannoma patients, which are currently unavailable for sporadic vestibular schwannomas.

**Abstract:**

Sporadic vestibular schwannomas (VSs) often exhibit slow or negligible growth. Nevertheless, some VSs increase significantly in volume within a few months or grow continuously. Recent evidence indicates a role of inflammation in promoting VS growth. Therefore, our study aimed to identify cytokines, which are associated with larger VSs. The expression of different cytokines in VS tumor samples and VS primary cultures was investigated. Additionally, the concentration of cytokines in cell culture supernatants of VS primary cultures and cerebrospinal fluid (CSF) of VS patients and healthy controls were determined. Correlation analysis of cytokine levels with tumor volume, growth rate, Koos grade, age, and hearing was examined with Spearman’s-rank test. The mRNA expression of CC-chemokine ligand (*CCL*) 18, growth differentiation factor (*GDF*) 15, and interferon regulatory factor 4 correlated positively with tumor volume. Moreover, the amount of GDF15 in the cell culture supernatant of primary cells correlated positively with tumor volume. The concentrations of the cytokines CCL2, CCL5, and CCL18 and transforming growth factor beta (TGFB) 1 in the CSF of the patients were significantly different from those in the CSF controls. Inhibition of immune cell infiltration could be a putative approach to prevent and control VS growth.

## 1. Introduction

Vestibular schwannoma (VS) is a benign tumor arising from the vestibular part of the eighth cranial nerve [1] and is the most common tumor of the cerebellopontine angle [2,3]. With the increased use of contrast-enhanced magnetic resonance imaging (MRI), VS has been diagnosed more frequently. Sporadic tumors are usually localized unilaterally, whereas VS associated with neurofibromatosis type 2-related schwannomatosis (NF2) occurs bilaterally [1,4].

Although approximately 50% of sporadic VS do not increase in size, there are tumors which show a rapid size progression [4]. This can lead to the displacement of adjacent cerebral structures and brainstem compression [5]. Symptoms such as hearing loss, unilateral tinnitus, and vertigo are frequently observed. So far, little has been known about the causes and mechanisms of VS size progression. Moreover, there is no established pharmacological therapy to prevent growth.

Besides monitoring the progression of VS with MRI (wait and scan), there are currently two therapeutic options [6]. One option is radiosurgery, and the other is resection. The size of the tumor is crucial for the decision of the procedure [6]. However, because VSs often have incalculable growth rates, the optimal time for therapy is difficult to estimate [7]. As tumors increase in size, the risk of a worse postoperative outcome for the auditory and facial nerve also increases [6]. There are also risks associated with both VS treatments, as hearing function can be affected, or other cranial nerves, especially the facial nerve, can be damaged [3].

In recent years, the importance of research on inflammatory processes and the influence of immune cells on VS progression has increased. A previous analysis showed that the majority of proliferating cells in the progressive VS were allograft inflammatory factor 1 (AIF1)-positive macrophages [5], which is a marker for the macrophages of the brain (microglia) [8]. In addition, higher mRNA levels of the macrophage markers *CD68* and *CD163* were detected in large, as well as in fast-growing, VSs [1,9]. In addition, rapidly growing VSs showed higher expression of macrophage colony-stimulating factor (M-CSF) [7]. Among these, CD68 was found on all macrophages and monocytes [10], whereas CD163 is a high-affinity scavenger receptor and a marker of tumor-associated M2 macrophages [11]. These have anti-inflammatory properties and promote tumor growth [11]. M-CSF is associated with higher macrophage activity and tumor progression [7], and it induces the differentiation of macrophages into M2 macrophages [12]. These macrophages exhibit tumor-promoting properties, such as suppression of the immune response, promotion of size growth, and angiogenesis in tumors [13].

In other tumors, cytokines are associated with the recruitment of macrophages from the peripheral blood, and their polarization and activation to form tumor-associated macrophages (TAMs). CC-chemokine ligand (CCL) 2, CCL5, and CCL18 and transforming growth factor beta (TGFB) 1 secreted by tumor cells have been described to have chemotactic effects on macrophages [14,15,16,17]. Positive correlations with the number of TAMs have been demonstrated for the expression of CCL2 and CCL5 [18,19]. Interferon regulatory factor (IRF) 4 and TGFB1 stimulate the polarization of macrophages to M2 macrophages through the activation of interleukin (*IL*) 4 and *IL10* genes [20,21]. Growth differentiation factor (GDF) 15 is described to be secreted by tumor cells, but also by TAMs. GDF15 was also shown to polarize macrophages towards the M2 subtype [22]. These mechanisms of macrophage attraction and polarization, as well as their role in tumor size progression, known for other tumor entities have not yet been investigated for VS.

Numerous substances targeting TAMs have been developed for treating malignant tumors, employing diverse therapeutic approaches. Firstly, the M-CSF receptor on macrophages can be directly inhibited by antibodies, leading to a reduction in macrophage infiltration into the tumor [23,24]. Secondly, the attraction and polarization of macrophages can be prevented by inhibiting cytokines or their receptors. Promising results have been observed in cancer patients’ treatment with Carlumab, an anti-CCL2 antibody, or Maraviroc, a CCR5 antagonist [25,26,27,28].

Therefore, the aim of this exploratory study was to elucidate the mRNA levels and concentrations of immunomodulatory cytokines in VS tumor samples, VS primary cultures, and cerebrospinal fluid (CSF) of VS patients for an understanding of macrophage recruitment as well as their polarization and activation into TAMs. Insight into the interaction between tumor cells and immune cells could lead to (i) a more thorough understanding of the progression of VSs and (ii) the identification of new drug targets against VS growth as there is currently no medical therapy option for VSs. A further aim of our study was to compare the mRNA level of cytokines in VSs with the mRNA level of corresponding vestibular nerves, as well as the cytokine concentrations in CSF of VS patients with the CSF cytokine concentration in controls, in order to investigate the influence of the immunomodulatory cytokines on the development of VS.

## 2. Materials and Methods

### 2.1. Study Design, Ethics, and Tumor Volumetry

**Figure 1:**
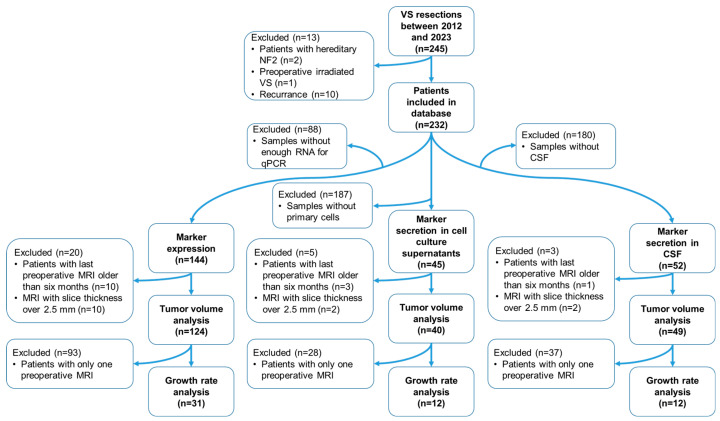
Workflow of the study. Of the 232 patients in the database, not all bio-material was available from each patient. In 176 patients, at least mRNA, primary culture, or CSF was present. mRNA analysis was obtained for 144 tumor samples, correlation with tumor volume could be obtained in 124 samples, and correlation with growth rate was found in 31 tumor samples. Quantification of cytokines in cell culture supernatants (CCSs) of primary cultures was performed in 45 samples, correlation with tumor volume was possible in 40 samples, and correlation with growth rate was possible in 12 samples. Concentration analysis of cytokines in CSF was feasible in 52 samples, whereby a correlation with tumor volume in 49 samples and a correlation with growth rate in 12 samples was obtained. Abbreviations: CSF, cerebrospinal fluid; MRI, magnetic resonance imaging; NF2, NF2-related schwannomatosis; qPCR, quantitative real time polymerase chain reaction; VS, vestibular schwannoma.

The database included 232 consecutive patients with sporadic VS from 2012 to 2023 who were at least 18 years old at the time of surgery and had at least one preoperative cranial MRI scan, acquired less than 6 months before the operation. Patients with recurrence, radiation, or NF2 diagnosis were excluded from the study (Figure 1) [9]. Data from 175 patients were used for the experimental analyses.

Preoperative tumor volume was determined by preoperative MRI as described in Leisz et al. [9]. For tumor volume and growth rate determination, only MRIs with a slice thickness of 2.5 mm or less were considered, resulting in tumor volume determination from 155 patients. To determine the growth rate, at least two preoperative images were required that had a slice thickness less than 2.5 mm and were at least 6 months apart, resulting in a growth rate determination from 39 patients. The MRI image history was used to calculate the absolute annual growth rate as follows:

$$\frac{tumor\;volume\;MRI\;time\;point\;2-tumor\;volume\;MRI\;time\;point\;1}{\left(MRI\;time\;point\;2-MRI\;time\;point\;1\right)/365}$$

The relative annual growth rate was calculated using the MRI images as follows:

$$\frac{tumor\;volume\;MRI\;time\;point\;2/tumor\;volume\;MRI\;time\;point\;1}{(MRI\;time\;point\;2-MRI\;time\;point\;1)/365}$$

In addition to the tumor volume, the VSs were classified according to the Koos grade, considering the extent of the VS in the cerebellopontine angle and the relation to the brain stem. Positive votes (approval numbers 2020-122 and 2021-101) of the ethics committee of the medical faculty of the Martin Luther University Halle-Wittenberg were obtained. The study was in accordance with the criteria of the Declaration of Helsinki. Written informed consent was obtained from each patient prior to surgery, which included the collection of patient data from medical records, the usage of preoperative MRI images, surgical specimens, and CSF. CSF samples from neurological patients provided a control group for the CSF concentration of cytokines. Patients were excluded from the control group if they had a disorder of the blood–brain barrier, an underlying inflammatory disease, a tumor, or a degenerative brain disease in their medical history. Hearing class was determined preoperatively from all VS patients according to the American Academy of Otolaryngology-Head and Neck Surgery (AAO-HNS) classification [29], as previously described in Rahne et al. [30]. The hearing class was determined on the basis of speech intelligibility (Word recognition score 40SL) and tone threshold audiometry (4 pure tone audiometry 0.5, 1, 2, 3 kHz). In addition, a fifth category DS (surditas for deafness) was defined for preoperatively deaf patients.

### 2.2. Nerve Samples, DNA Extraction, and Genotyping

The nerve sections originate from the vestibulocochlear nerve. Nerve samples were obtained from 10 patients during the vestibular schwannoma surgery. DNA was isolated from these nerves, as well as from the corresponding tumor samples, using the Qiagen AllPrep DNA/RNA Micro Kit (Qiagen, Hilden, Germany). Then, 350 µL RLT buffer plus one percent beta-mercaptoethanol was added to the samples. The samples were then ground in the Tissue Lyser for 2 × 2 min at 50 Hz. The samples dissolved in the buffer were pipetted onto a DNA column to bind the DNA. After washing the columns twice, the DNA was dissolved in EB buffer, which was heated to 70 °C in advance. The tumor samples and the corresponding nerves were genotyped using Infinium EPIC Array v2.0 (Life&Brain Genomics, Bonn, Germany). All tumors could be identified as schwannomas with a calibrated score > 0.9 using DNA methylation-based classification [31] (www.molecularneuropathology.org, brain_classifier_v12.8; last accessed on 4 January 2024).

### 2.3. CSF Samples

The patients’ CSF was collected intraoperatively after opening the basal cistern. The CSF of the control group was obtained to exclude neurological diseases, and no abnormalities were found in the CSF. According to standardized procedures, 10–12 mL of CSF was collected in sterile polypropylene tubes and centrifuged at 300× *g* and 4 °C for 10 min to remove cells. The supernatant was aliquoted and stored at −80 °C until measurement.

### 2.4. RNA Extraction from Tumors and Corresponding Nerve Samples and Reverse Transcription into cDNA

RNA was isolated from VS tumor samples and corresponding vestibular nerve samples and reverse transcribed into cDNA as described in Leisz et al. [9]. Briefly, the Qiagen AllPrep DNA/RNA/Protein Mini Kit (Qiagen, Hilden, Germany) was used for RNA isolation. The cDNA synthesis was performed using the RevertAid First Strand cDNA Synthesis kit (Thermo Fisher Scientific, Waltham, MA, USA).

### 2.5. RNAseq Analysis of Tumor and Corresponding Nerve Samples

For nerve and tumor tissue samples, RNA was also isolated as described in Section 2.2. Ten tumor and nerve pairs were selected for bulk RNA sequencing (RNAseq). The RNA of tumor samples was also used for qPCR analysis. The RNAseq data were obtained from 1 µg of RNA using the Illumina Novaseq6000 system (Novogene, Cambridge, UK). Subsequently, the data were further processed using the Galaxy Server (https://usegalaxy.eu/, accessed on 13 December 2023) essentially as described in Wieland et al. [32]. Mapping of reads to the human genome version HG38 was performed using Hisat2, and read quantification was performed using featureCounts. The RNAseq data of the tumors were compared with the corresponding nerve sample using Wilcoxon matched-paired signed rank test and visualized graphically using GraphPad Prism version 10 (GraphPad Software, Boston, MA, USA).

### 2.6. Isolation of Primary Cultures from Cavitron Ultrasonic Surgical Aspirator Filter-Obtained Tissue

Primary cultures were obtained from the Cavitron Ultrasonic Surgical Aspirator (CUSA) filter-obtained tissue. The isolation and characterization of primary cultures were performed as previously established in Leisz et al. [33]. In brief, tissue was washed with phosphate buffered saline without Ca^2+^ and Mg^2+^ (PBS; Thermo Fisher Scientific, Waltham, MA, USA), digested with an enzyme solution of collagenase and hyaluronidase (both enzymes Merck, Sigma-Aldrich, Darmstadt, Germany) for approximately 14 h, and then further digested with another enzyme solution (DNase and trypsin, Gibco, Thermo Fisher Scientific, Pittsburgh, PA, USA) for 5 min. The cells were now plated onto a 75 cm^2^ cell culture flask (Sarstedt, Nümbrecht, Germany) pre-coated with poly-L-lysine and poly-L-ornithine (both Merck, Sigma-Aldrich, Darmstadt, Germany). The isolated primary cultures were mainly S100-positive and CD56-positive cells (Table S1). S100 and CD56 served as markers for the identification of schwannoma cells [34,35].

### 2.7. Immunofluorescence Staining

To characterize VS primary cells, immunofluorescence analysis was used. Therefore, the chambers of a slide (4-well Tissue Culture Chambers, Sarstedt, Nümbrecht, Germany) were coated with poly-L-lysine and poly-L-ornithine, and primary cells were seeded on the slide. The slices were incubated for 24 h. Following washing with Hanks’ Balanced Salt Solution with Ca^2+^ and Mg^2+^ (Thermo Fisher Scientific, Waltham, MA, USA), the cells were fixed with ice-cold methanol. The slides were washed with PBS before the cells were blocked with a blocking buffer (PBS with 5% normal goat serum (Cell Signaling, Danvers, MA, USA) and 0.3% Triton X 100 (Carl Roth, Karlsruhe, Germany)). The cells were stained with primary antibodies against S100 and CD56 (Table S2) and incubated overnight at 4 °C. The slides were washed and stained with the secondary anti-mouse-antibody (Table S2). The slides were covered with a mounting medium containing DAPI (ImmunoSelect Antifading Mounting Medium DAPI, Dianova, Hamburg, Germany). The images were captured with a Keyence BZ-800E microscope (Keyence, Neu-Isenburg, Germany). The cells were quantified using the IdentifyPrimaryObjects tool of the CellProfiler software (version 4.2.4 (Broad Institute, Cambridge, MA, USA)). The proportion of S100^+^ and CD56^+^ in the total cell count, which was determined using the number of DAPI-positive cells, was calculated.

### 2.8. Obtaining Cell Culture Supernatants from Primary Cultures and RNA Isolation from Primary Cells

To obtain cell culture supernatants (CCSs), 4 × 10^5^ primary cells per well were seeded on a six-well plate in 3 mL DMEM (Dulbecco’s Modified Eagle Medium)/F-12 (Gibco, FisherScientific, Pittsburgh, PA, USA). The primary cells were incubated for 48 h at 37 °C at 5% CO_2_ in a humidified incubator. Subsequently, 2 mL of the CCS was collected and centrifuged at 300× *g* for 10 min at 4 °C to remove cells. The supernatant was transferred to a new tube and stored at −80 °C. Wells were washed twice immediately with ice-cold PBS. Then, cells were lysed directly by the addition of 350 µL LBP buffer (Macherey-Nagel, Düren, Germany), transferred to a tube, and stored at −20 °C. The NucleoSpin RNA Plus Kit (Macherey-Nagel, Düren, Germany) was used for the isolation of RNA from the cell lysate according to the manufacturer’s instructions.

### 2.9. Quantitative Real-Time PCR

Quantification of cytokine mRNA levels was performed by quantitative real-time polymerase chain reaction (qPCR), as described previously [9]. Specific forward and reverse primers (Table S3) were obtained from Invitrogen (Thermo Fisher Scientific, Waltham, MA, USA). The 2^−ΔΔCT^ method was used for the analysis, and glycerin aldehyde 3-phosphate dehydrogenase (*GAPDH*) served as constitutive housekeeping gene.

### 2.10. Enzyme-Linked Immunosorbent Assay

Quantification of cytokines in CCS and CSF was performed by using the Ella Enzyme-linked immunosorbent assay (ELISA) system (ProteinSimple, BioTechne, Minneapolis, MN, USA). A multi-analyte cartridge (ProteinSimple, BioTechne, Minneapolis, MN, USA) was used to determine the concentration of the cytokines CCL2, CCL5, and CCL18 in parallel. TGFB1 and GDF15 concentrations were detected on separate cartridges. After centrifugation (300× *g* for 5 min at 4 °C), CCS and CSF were diluted from 1:1.5 to 1:25 for concentration determination and activated in advance with 1N HCl for measurement of TGFB1, according to the manufacturer’s instructions. The cartridge was loaded with 50 µL of activated and diluted CCS or CSF per well. Total protein concentrations of CCS and CSF were determined using the Pierce BCA Protein Assay Kit (Thermo Fisher Scientific, Waltham, MA, USA) according to the manufacturer’s instructions. Concentrations of cytokines and total protein concentration were similarly determined in the CSF of a control group. The percentage of cytokine concentration of the total protein concentration was calculated.

### 2.11. Statistical Analysis

Initial exploratory correlations examined mRNA values or the concentration of immunomodulatory cytokines with tumor volume and growth rate. Correlations were assessed as shown in Table S4. A separate analysis was performed with a smaller number of patient samples in which a tumor growth rate was available. Spearman’s rank correlation was used due to the ordinal scaling of the AAO-HNS hearing classification and the non-normal distribution of the other parameters. For this purpose, the mRNA level data were normalized to the first patient sample by forming a ratio. For the ELISA data, the proportion of the total protein concentration was first determined and then normalized to the first patient sample as for the mRNA data. For further confirmation of the correlations with tumor volume, a linear regression analysis of the factors significantly correlated with tumor volume was performed.

To illustrate the results in the correlation analysis of mRNA levels in tumor samples, a non-parametric Wilcoxon signed-rank test was conducted. For this purpose, the results of the large (tumor volume > 7.5 cm^3^) and small (tumor volume < 0.4 cm^3^) VSs were examined comparatively with respect to the different markers. The differences between the groups were examined using Fisher’s exact test (gender) and Mann–Whitney test (hearing class, tumor volume, Koos grade, and age).

For comparisons between groups, the interquartile range (IQR) was used as a dispersion parameter. *p*-values of correlations and comparisons between controls, patients, and largest and smallest tumors were false discovery rate (FDR)-corrected for multiple comparisons [36].

The RNAseq data analysis as well as the creation of the corresponding graphs were performed with Prism version10 (GraphPad Software, Boston, MA, USA). All other analyses were calculated with R 4.0.5 [37].

## 3. Results

### 3.1. Cytokine mRNA Analyzed by qPCR Correlates Positively with Tumor Volume and Macrophage Markers but Not with Tumor Growth Rate

#### 3.1.1. Correlation Analysis of Investigated Markers with Tumor Volume in Tumor Samples from 144 VS Patients

**Figure 2:**
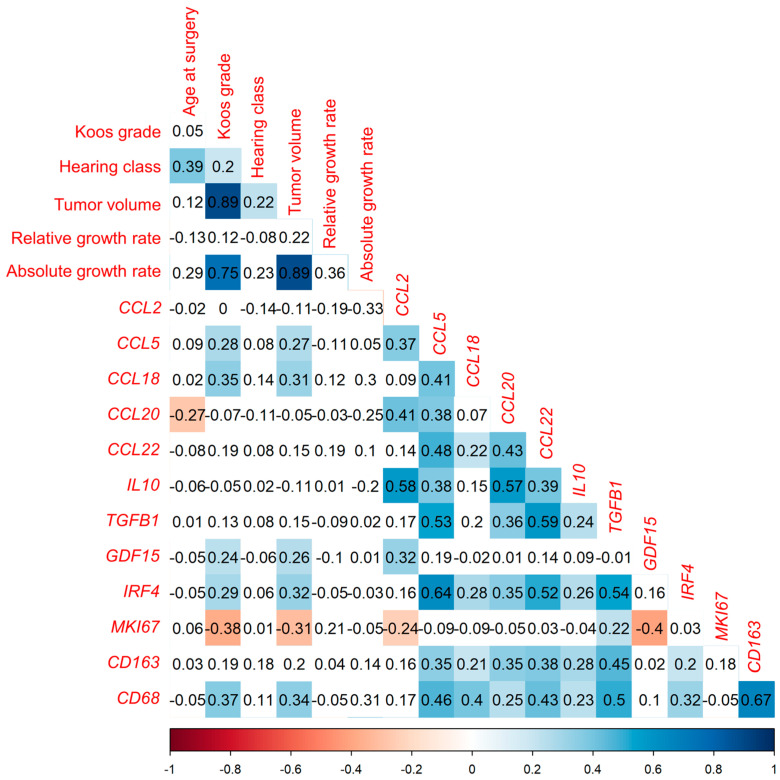
Correlation analysis of the indicated markers and clinical parameters in 144 VS tumor samples using Spearman’s rank test. Depicted is the correlation between the mRNA levels of the 10 examined markers and age as well as Koos grade of 144 patients, and the correlation of marker levels with hearing class in 143 patients and with tumor volume in 124 patients. The correlation coefficient r is shown. Significant negative correlations (*p* < 0.05) are plotted in red and significant positive correlations (*p* < 0.05) in blue. Non-significant correlations are plotted uncolored. *p*-values of correlations were FDR-corrected for multiple comparisons.

The correlation of the mRNA level analyzed by qPCR of eight immunomodulatory cytokines (*CCL2*, *CCL5*, *CCL18*, *CCL20*, *CCL22*, *IL10*, *TGFB1*, and *GDF15*), the transcription factor *IRF4*, the marker of proliferation Kiel 67 (*MKI67*), and two macrophage markers (*CD68*, *CD163*) with clinical parameters such as tumor volume (n = 124), growth rate (n = 31), relative growth rate (n = 31), hearing class (n = 143), Koos grade (n = 144), and age at surgery (n = 144) were investigated (Figure 2). For this purpose, a Spearman’s-rank test was utilized, and *p*-values of correlations were FDR-corrected for multiple comparisons. Of the 144 patients, there were 61 males and 83 females (Table S5). The median tumor volume was 2.1 cm^3^ (IQR 15.25). The mRNA levels of the cytokines *CCL5*, *CCL18*, and *GDF15*, as well as the transcription factor *IRF4*, correlated weakly positively with the tumor volume (Table S6). Furthermore, the mRNA levels of the macrophage marker *CD68* correlated weakly positively with the tumor volume. Moreover, these factors also correlated weakly positively with the Koos grade. In addition, the mRNA levels of the cytokine *CCL22* correlated very weakly positively with the Koos grade. Most mRNA levels of the cytokines correlated weakly or moderately with the macrophage markers *CD68* and *CD163*. An exception to this is the mRNA level of *GDF15*. The mRNA level of *GDF15* does not correlate with the mRNA levels of the macrophage markers. Apart from the *GDF15* mRNA level, the mRNA levels of the investigated cytokines correlated with each other. The mRNA levels of the analyzed cytokines did not correlate with the growth rate or the relative growth rate.

Linear regression analysis of parameters correlating significantly with tumor volume in correlation analysis was performed. Of the markers that correlate significantly with tumor volume, growth rate (estimate 2.940, standard error 0.434) and mRNA levels of *IRF4* (estimate 1.063, standard error 0.358) and *CD68* (estimate 0.545, standard error 0.208) were found to have a significant positive association with tumor volume in the regression analysis (Table S7). In this analysis, the adjusted R-squared was 0.898, and the F statistic was significant (*p* < 0.001).

#### 3.1.2. Increased CCL18 and IRF4 mRNA Levels in VSs with Large Tumor Size

To analyze whether the different marker levels investigated in Figure 2 depended on tumor size, Wilcoxon signed-rank tests were performed. Two groups were selected from the cohort of tumors, with one group containing the 20 smallest tumors and the other group containing the 20 largest tumors. This resulted in the small tumors having a tumor volume smaller than 0.4 cm^3^ and the large tumors having a tumor volume larger than 7.5 cm^3^. In both cohorts, the sex distribution was almost equal (small VS: 8 males, 12 females, median 51, IQR 16; large VS: 10 males, 10 females, median 49, IQR 20.25; Table S8). The median tumor volume in the group of small tumors was 0.3 cm^3^ (IQR 0.17 cm^3^); the median tumor volume in the group of large tumors was 12.5 cm^3^ (IQR 7.59 cm^3^).

**Figure 3:**
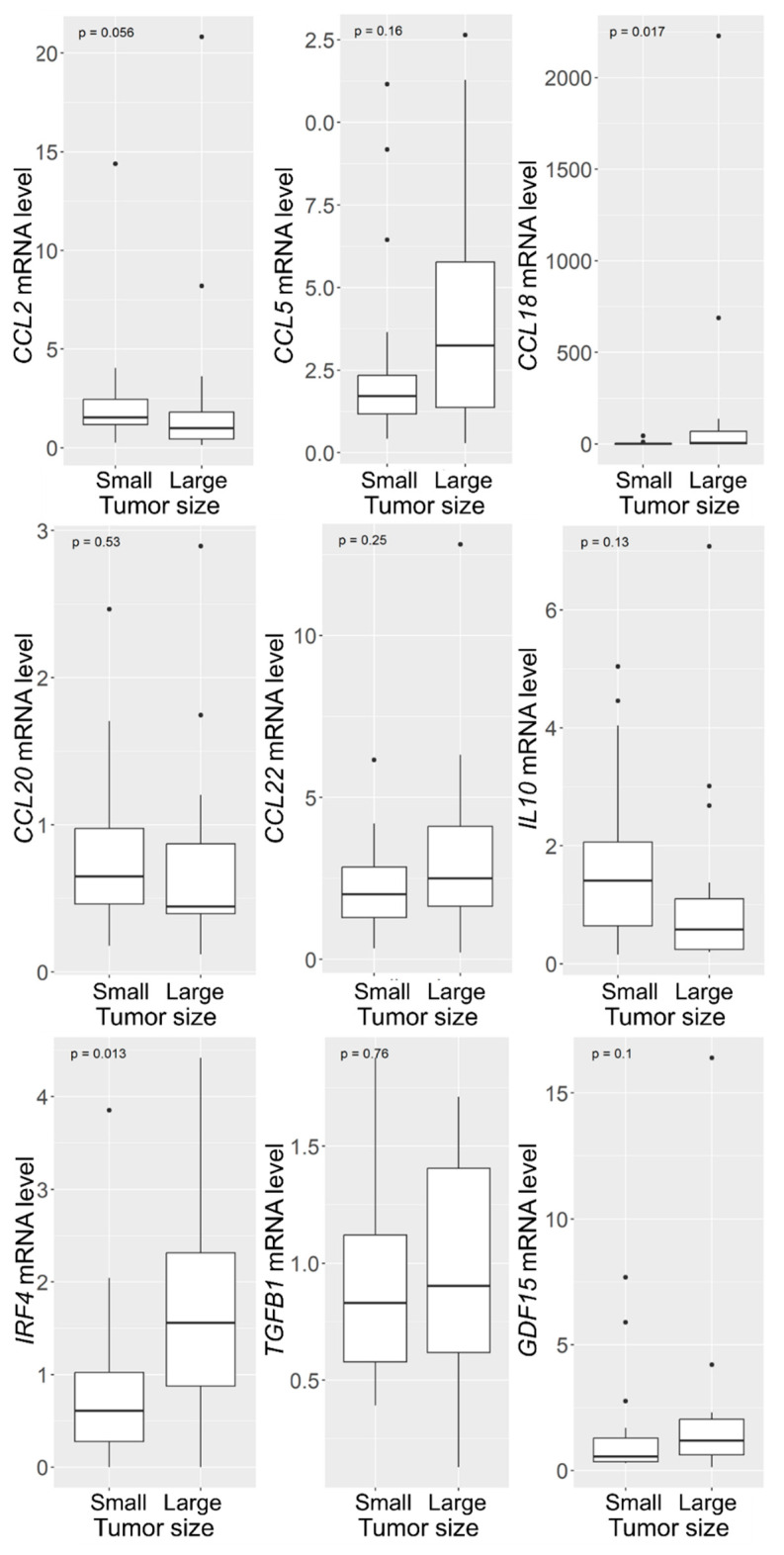
Box-plots of the studied markers in 20 small and 20 large VSs. The box represents the values from the first to the third quartile. The whiskers from the box extend to 1.5 times the interquartile range. *p*-values from non-parametric Wilcoxon signed-rank test are presented.

There was no significant difference in patient age and gender between the two groups (Table S8). Koos grade and hearing class were higher in the larger tumor group than in the smaller VS group. The analysis of the investigated markers in the large and small VS revealed a statistically significant difference in two markers (Figure 3). The mRNA levels of the cytokine *CCL18* were significantly higher in large tumors compared to small tumors (*p* = 0.017), with the median of the large tumors being 5.26 and the median of the small tumors being 0.69 (Table S9). The levels of the transcription factor *IRF4* were increased two-fold in the large VS (median = 1.56) compared to mRNA levels in the small tumors (median = 0.61, *p* = 0.013). In addition, the mRNA levels of *CCL2* showed a tendency to be higher in the small tumors than in large VS (*p* = 0.056). The median of the small tumors increased approximately two-fold compared to the small tumors (small VS median = 1.54, large VS median = 0.98).

### 3.2. Differences in RNA Transcription Levels Investigated Using RNAseq between 10 VS Tumors and the Corresponding Vestibular Nerves

The RNA reads of immunomodulatory cytokines, their receptors, schwannoma cell markers, and macrophage markers were determined in 10 tumor samples and the 10 corresponding nerves using RNAseq. Half of the patients were male (n = 5) and the other half female (n = 5, Table S10).

**Figure 4:**
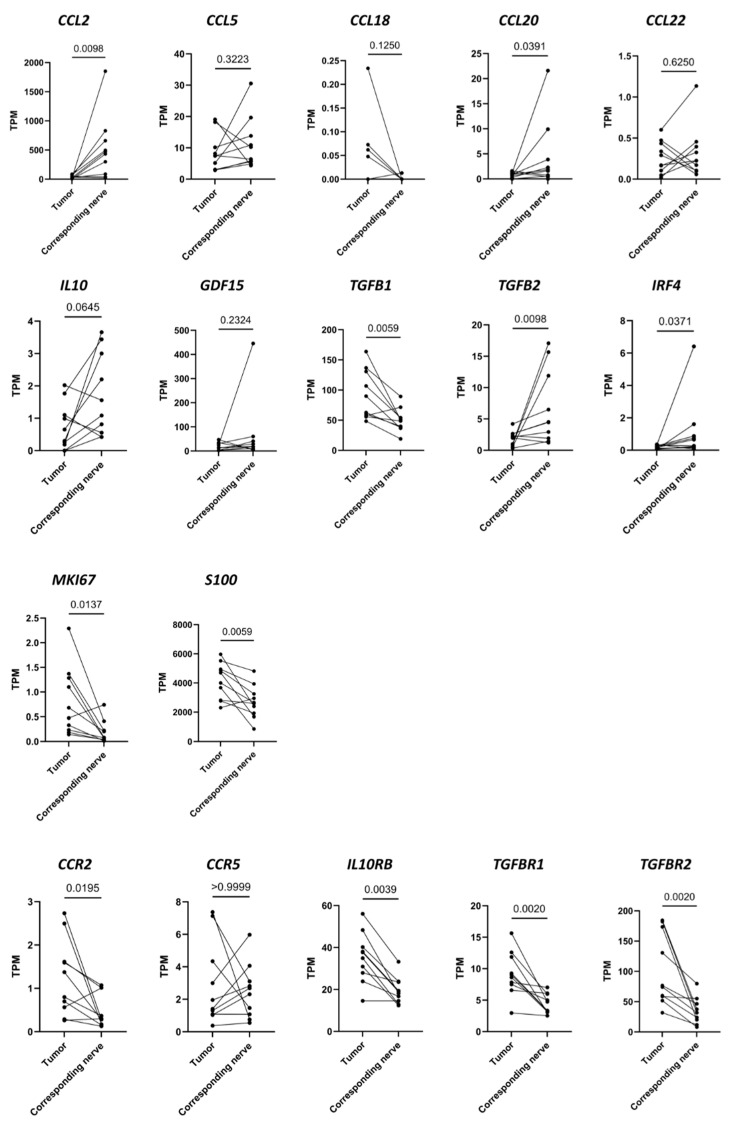
The transcripts per million (TPM) of the indicated genes in 10 tumor samples and the 10 corresponding samples of vestibular nerves are shown. The TPM of the tumor is connected to the TPM of the corresponding nerve from the same patient by a line. The mRNA in the tumors was compared with the mRNA in the nerves using a Wilcoxon matched-paired signed rank test. The corresponding *p*-values are presented.

Some of the cytokines and receptors examined showed differential expression between the tumors and the vestibular nerves (Figure 4). The mRNA of the cytokines *CCL2*, *CCL20*, and *TGFB2*, as well as the mRNA of the transcription factor *IRF4,* had higher expression in the nerves than in the tumors. The mRNA levels of *TGFB1*, *MKI67*, and *S100* were higher in the tumors than in the vestibular nerves. In addition, the mRNA of the receptors *CCR2*, *IL10RB*, *TGFBR1*, and *TGFBR2* were expressed at higher levels in the tumors compared to the nerves.

### 3.3. TGFB1 and GDF15 Concentration in CCS Correlates Positively with Tumor Volume

#### 3.3.1. Concentrations of the Investigated Cytokines in CCS of VS Primary Cultures

**Figure 5:**
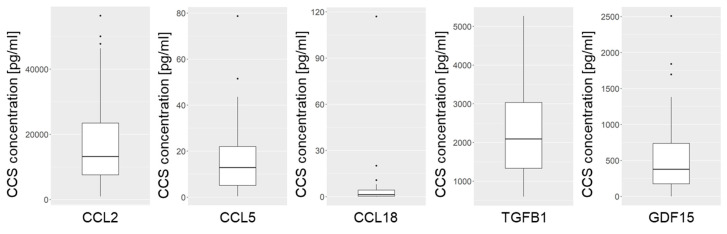
Concentrations of the investigated cytokines in the CCS of 45 primary cultures from patients with sporadic VSs. The borders of the box range from the 25th to 75th percentiles, and outliers are visualized as dots. The median is represented by the crossbar.

The concentration of five immunomodulatory cytokines (CCL2, CCL5, CCL18, TGFB1, and GDF15) was determined in the CCS of 45 VS primary cultures. The transcription levels of these cytokines in the primary cells were examined subsequently. The samples for this study were from 18 male and 27 female patients (Table S11). The median tumor volume was 2.47 cm^3^ (IQR 3.44 cm^3^). Isolated cells were positive for S100 and CD56 (Table S1), as described earlier for schwannoma cells [34,35]. All cytokines examined could be detected in the CCS of VS primary cultures, although the concentrations varied widely (Figure 5, Table S12). The concentration of CCL2 in the CCS of the primary cultures was the highest, with a median concentration of 13,199 pg/mL (950–56,407 pg/mL). The range of concentrations was also high for CCL2. With a median of 2092 pg/mL (602–5268 pg/mL), the TGFB1 concentration in the CCS was second highest. A concentration of 378 pg/mL (0.4–2509 pg/mL) was detected for GDF15. The concentration of CCL5 was noticeably lower, with a median of 12.9 pg/mL (0.4–78.7 pg/mL). However, the concentration of CCL18 in the CCS was the lowest (1.36 pg/mL; 0.008–117 pg/mL).

#### 3.3.2. Correlation Analysis of Cytokine mRNA Levels and Secretion in VS Primary Cultures

**Figure 6:**
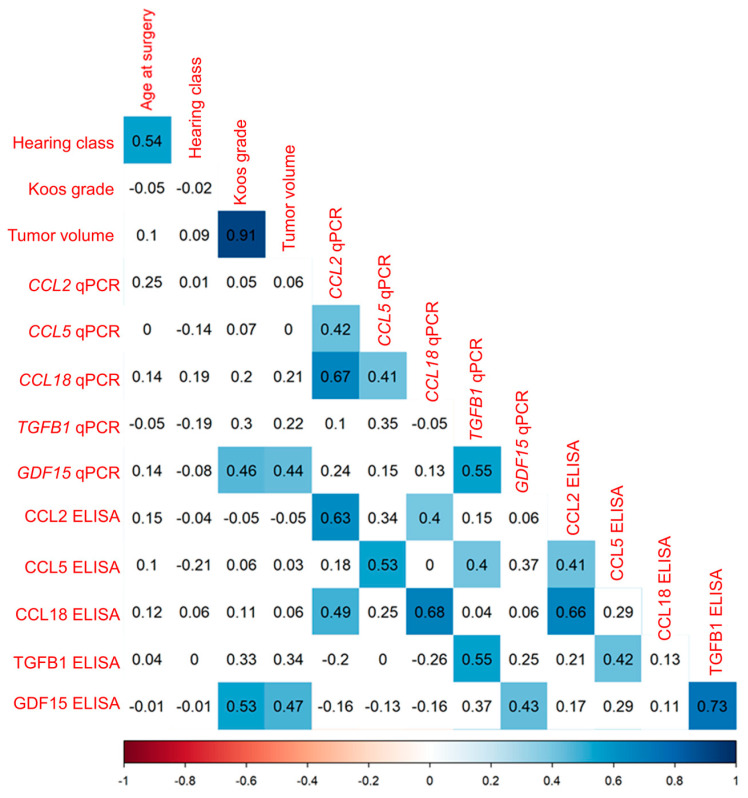
Correlation analysis of primary culture mRNA levels (with suffix qPCR) and concentration (with suffix ELISA) of various cytokines. Illustrated is the correlation of the mRNA levels and concentration of the investigated markers with one another, with age, hearing class, and Koos grade in 45 patients and with tumor volume in 44 patients. The correlation coefficient r is displayed in each panel. Areas plotted blue (*p* < 0.05) indicate significant positive correlations. Non-significant correlations are plotted uncolored. *p*-values of correlations were FDR-corrected for multiple comparisons.

The transcription levels of the five cytokines *CCL2*, *CCL5*, *CCL18*, *TGFB1*, and *GDF15* in VS primary cultures were correlated with their concentration in CCS of VS primary cultures. These data were correlated with clinical data, such as patient age and Koos grade in 45 patients, hearing class in 43 patients, and tumor volume in 40 patients. The mRNA levels of the investigated cytokines were moderately to strongly positively correlated with the concentration of the corresponding cytokine in the CCS (Figure 6, Table S13). The tumor volume was weakly to moderately positively correlated with the *GDF15* transcript levels in the primary cells, as well as the GDF15 concentrations in the CCS. Some of the mRNA levels and concentrations of the cytokines correlated positively among each other.

A linear regression analysis was performed for the markers that correlated significantly with tumor volume (Table S14). Among these markers, only the mRNA amount of *GDF15* correlated with tumor volume (estimate 1.637, standard error 0.282). The adjusted R-squared was 0.001919, and the *p*-value of the F statistic was significant (*p* < 0.001).

### 3.4. Distinct Cytokine Concentrations in CSF of VS Patients Compared to Control Group

#### 3.4.1. Concentration of Cytokines in CSF of VS Patients Compared to Control Group

**Figure 7:**
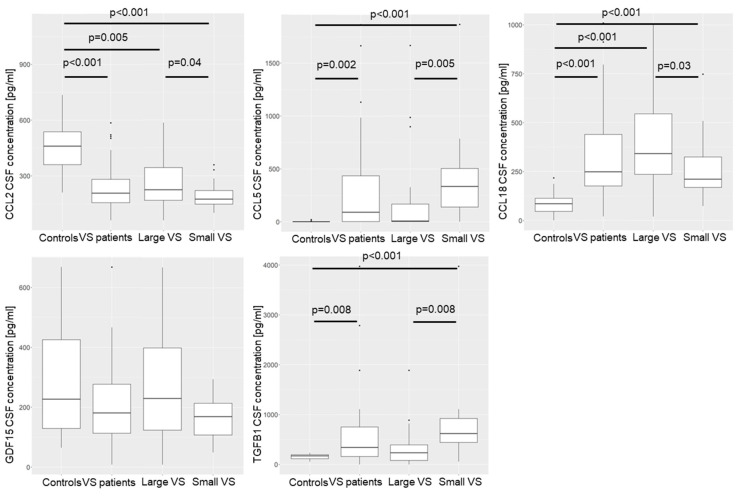
The concentration of the cytokines CCL2, CCL5, CCL18, GDF15, and TGFB1 in the CSF of a control group (n = 14) was compared with the concentration in the CSF of 52 patients, as well as with the concentration in the CSF of the 20 largest and 20 smallest VSs. A Wilcoxon signed-rank test was performed to compare the groups. The median concentration is marked by the crossbar. The boundaries of the boxes represent the interquartile range, and the whiskers represent 1.5 times the interquartile range. Outliers are presented as dots. *p*-values of correlations were FDR-corrected for multiple comparisons.

The concentrations of the cytokines CCL2, CCL5, CCL18, TGFB1, and GDF15 were determined in the CSF of 52 VS patients and 14 healthy controls. The concentration of cytokines in the control group was compared with the concentration in the patient group, as well as with the concentration in small-VS large tumors. Two groups were formed from the cohort of tumors, with one group containing the 20 smallest tumors and the other group containing the 20 largest tumors. The small tumor group was also compared with the large tumor group. In the patient group and the large and small tumors, respectively, the genders were evenly distributed (Table S15). However, there were more females (9 patients) than males (5 patients) in the control group (Table S16). The median tumor volume of the patients was 2.8 cm^3^ (IQR 4.55 cm^3^; Table S15). The median tumor volume of small VSs was 0.8 cm^3^ (IQR 1.09 cm^3^), and the median tumor volume of large VSs was 7.3 cm^3^ (IQR 4.03 cm^3^). The concentration of the five cytokines in the CSF of the patients was at similar levels (Table S17). Independent of the tumor volume, the concentration of CCL2 in the CSF of the patients was lower than in the CSF of the control group (Figure 7). The opposite was observed for CCL5, CCL18, and TGFB1. Compared to the control group’s CSF, there was a higher concentration in the CSF of the patients. Interestingly, the concentrations of the cytokines CCL5 and TGFB1 were higher only in the patients with small tumors than in the healthy control group (Table S16).

Differences were also observed between the large and small tumors. The concentration of CCL2 and CCL18 was higher in the CSF of the patients with large tumors than the concentration in the CSF of the patients with small tumors. In contrast, the concentration of CCL5 and TGFB1 in the CSF of the patients with a small VS was higher than in the CSF of the patients with a small VS.

#### 3.4.2. Decreased CCL5 and TGFB1 CSF Concentration with Increasing Tumor Volume of VS

**Figure 8:**
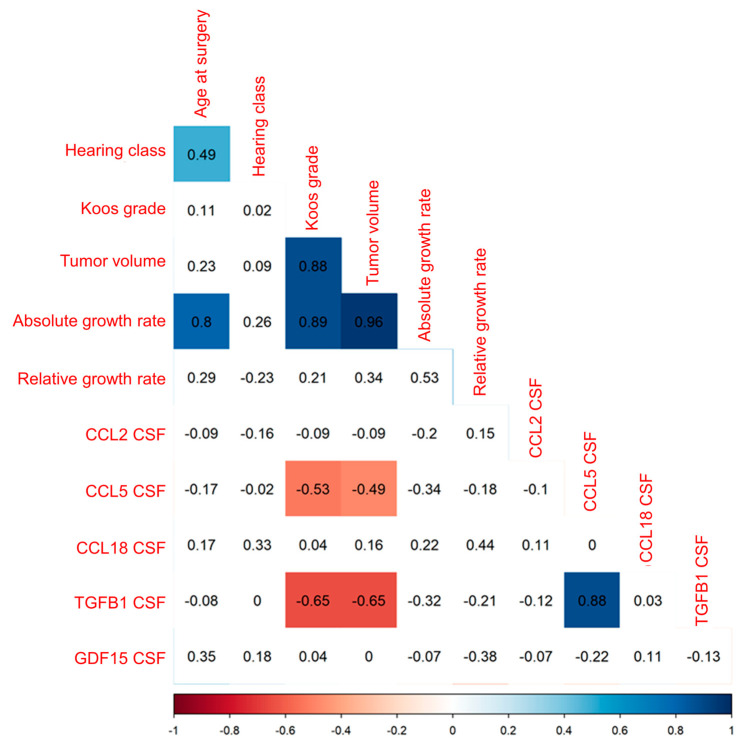
Illustrated is the correlation of the concentration of CCL2, CCL5, CCL18, TGFB1, and GDF15 in the CSF of 52 patients with clinical parameters, such as age, hearing class, Koos grade, and tumor volume, as well as the correlation of the concentration of the five cytokines in the CSF of 12 patients with growth rate and relative growth rate. Spearman’s r is plotted for each correlation. Significant positive correlations (*p* < 0.05) are colored blue, and significant negative correlations are colored red. Non-significant correlations are plotted colorless. *p*-values of correlations were FDR-corrected for multiple comparisons.

The correlation of the concentration of the five cytokines CCL2, CCL5, CCL18, TGFB1, and GDF15 in CSF of VS patients with Koos grade and patient age in 52 patients and with preoperative hearing class and tumor volume in 49 patients, as well as the correlation of the five cytokines with absolute and relative growth rate in 12 patients, was analyzed (Figure 8). CCL5 and TGFB1 CSF concentration was moderately to strongly negatively correlated with tumor volume, as well as with Koos grade (Table S18).

A linear regression analysis of growth rate and CSF cytokine concentrations significantly correlating with tumor volume revealed a significant association for growth rate (estimate 1.986, standard error 0.166) only (Table S19). The F statistic was significant for the analysis, and the adjusted R-squared resulted in a value of 0.9395.

## 4. Discussion

In recent years, there has been increasing interest in the role of TAMs in VS progression. Leisz et al. and Hannan et al. demonstrated a correlation of TAMs with tumor volume and growth rate [1,9,38]. This is congruent with our results. In many malignant tumors, such as breast, colorectal, and ovarian cancers, TAMs play an important role in tumor progression [39,40,41,42]. In carcinomas, macrophages are known to be attracted to the tumor by various cytokines produced by tumor cells (but also by macrophages) and polarized to the tumor growth-promoting M2 subtype [42,43,44]. Thus, the influence of macrophages and immunomodulatory cytokines on tumor progression can be assumed. Cytokines, including CCL2, CCL5, CCL18, TGFB1, and GDF15, are produced by tumor cells and attract immune cells, such as macrophages and lymphocytes, from the blood and bone marrow into the tumor microenvironment [14,16,22,42,45,46,47].

These cytokines were expressed in tumor samples and secreted by the primary VS cells in our study. Taken together with the mRNA levels of the cytokines’ receptors, this might suggest that the cytokines influence the attraction of immune cells in the VS and, thus, might influence the tumor progression. Macrophages could be polarized to the tumor growth-promoting M2 subtype in the tumor environment by cytokines, such as CCL2, CCL5, CCL18, and TGFB1 [48,49,50,51] and the mRNA levels of the transcription factor *IRF4* [52,53]. The presence of M2 macrophages in VS has already been demonstrated in some previous studies [1,54]. In addition, M2 macrophages were recently detected in immunohistological sections in Leisz et al. [9]. With increasing tumor volume, an increased number of macrophages was determined. The mRNA levels of the cytokines *CCL5*, *CCL18*, and *GDF15*, as well as the transcription factor *IRF4*, showed a positive correlation with the tumor volume. Furthermore, the cytokines CCL2, CCL5, CCL18, and TGFB1 were secreted by the VS primary cells. Additionally, a differential expression of the cytokine genes *CCL2*, *CCL20*, *TGFB1*, and *TGFB2*; the transcription factor gene *IRF4*; and the receptor genes *CCR2*, *IL10RB*, *TGFBR1*, and *TGFBR2* was identified in tumor tissue and vestibular nerve tissue. These results could indicate a polarization of TAMs to the M2 subtype by cytokines in the VS, but in addition, a potential influence of cytokine concentrations on the development of VSs. The M2 subtype is associated with increased tumor cell proliferation, metastasis, and vascularization in malignant tumors [22,55,56,57,58,59]. In malignant tumors, the polarized M2 macrophages produce and secrete cytokines such as CCL18, CCL20, CCL22, IL10, TGFB1, and GDF15 [22,55,60,61,62]. These cytokines are known to mediate tumor progression, angiogenesis, and, in the case of malignant tumors, invasion and metastasis through various mechanisms [22,55,56,57,58,59]. The immune escape of tumor cells can also be induced by CCL18 and TGFB1 [63,64]. The mRNA levels of *CCL18* and *GDF15* were positively correlated with tumor volume in the tumor samples, and the other cytokines were also expressed in the tumor samples. In malignant tumors, the macrophages are first attracted by the interaction of various cytokines and then polarized to the M2 subtype. Subsequently, tumor progression is promoted by the production of further cytokines and growth factors originating from the M2 macrophages in malignant tumors.

To date, little is known about the influence of cytokines on the development and progression of benign tumors such as VSs. Löttrich et al. detected TGFB1, TGFBR1, and TGFBR2 expression in VS [65], whereas Brieger et al. detected no TGFB1 expression by immunohistochemistry [66]. Increased mRNA levels of *TGFB1* were detected in VS tissue compared with normal vestibular tissue [67], and Bizzarri et al. detected the secretion of TGFB1 by VS cells [68]. Weerda et al. hypothesized an autocrine growth factor secretion of TGFB1 by VS cells [69]. Our data suggest that *TGFB1* mRNA is not only present in the tumor but is indeed secreted by the tumor cells and could play a role in the development of VSs from vestibular nerves, which has not yet been investigated in VSs. In addition to the increased levels of a macrophage marker in large tumors, our study suggests an association between the mRNA levels of the cytokines *CCL5*, *CCL18*, and *GDF15*, as well as the transcription factor *IRF4*, and VS tumor volume, which has not yet been investigated in VSs. Moreover, an interaction of cytokines and macrophages could be suggested by the correlation of macrophage marker levels with the mRNA levels of most cytokines. However, the regression analysis only showed a significant correlation between tumor volume and tumor growth rate as well as *IRF4* and *CD68* mRNA levels with growth rate having the strongest influence on tumor volume. Nevertheless, the adjusted R-squared determined in this analysis was very high. This could indicate that the other significant correlations with tumor volume in the previous correlation analysis could be explained by the associations of growth rate and *IRF4* mRNA levels in the regression. This is also consistent with the results, as *IRF4* is a transcription factor promoting the polarization of macrophages to the M2 subtype. For benign VS, there are few studies on the cytokine concentrations in cell culture supernatants of schwannoma cells. Stankovic et al. and Dilwali et al. demonstrated the secretion of CCL2 and CCL22 by VS primary cells into cell culture supernatants [70,71]. The primary VS cells likewise secreted the cytokine CCL2. In addition to CCL2, the examination of our cell culture supernatants of VS primary cultures suggests that vestibular schwannoma cells produce the cytokines CCL5, CCL18, TGFB1, and GDF15, which has not yet been investigated for VSs. The concentration of the cytokine GDF15 correlated with tumor volume, suggesting that this cytokine could have an impact on tumor progression. Unfortunately, these significant correlations were not confirmed in the regression. However, a significant association with the *GDF15* mRNA levels was calculated. Due to the highly adjusted R-squared, it may be concluded that the VS primary cells transcribe *GDF15* mRNA, which could lead to the secretion of the protein into the CCS.

Considering these results for benign VSs, it can be concluded that a mechanism similar to that in malignant tumors may be possible. These data suggest possible mechanisms by which tumor cells might influence the tumor microenvironment. Accordingly, macrophages could be attracted and polarized by these cytokines produced by tumor cells. In the polarization to the M2 subtype, the transcription factor *IRF4* detected in this study might be involved in macrophage polarization as well. An immune escape of VS triggered by cytokines, such as CCL18, also needs to be evaluated. However, the detection of cytokines solely does not allow for a conclusion on the relationship between these cytokines and tumor cells as well as macrophages. In addition, these data cannot be used to infer the cells that produce and secrete the cytokines investigated with qPCR. Moreover, it is possible that CCL2, CCL5, CCL18, TGFB1, and GDF15 are also produced by cells other than the VS tumor cells. Further studies are needed to determine the influence of macrophages on the tumor cells and whether cytokines are also produced by the TAMs.

To date, there have been few analyses of the concentration of immunomodulatory cytokines in the CSF of VS patients. The study by Nisenbaum et al. showed lower CCL2 levels in the CSF of patients compared to a healthy control group [54]. This finding is congruent with our results, as we investigated a lower concentration of CCL2 in the CSF of patients compared to control CSF. The decreased concentration of CCL2 in the CSF of the patients compared to the control CSF is consistent with the findings of the RNA analysis of tumor tissue and corresponding vestibular nerves and might be explained by the fact that mRNA levels of *CCL2* were lower in the tumor tissue than in the corresponding vestibular nerves. In contrast to *CCL2*, the CCL2 receptor *CCR2* exhibited higher expression levels in tumors than in vestibular nerve tissue. No other cytokines have yet been investigated in the CSF of patients with VSs. In contrast, there are several studies on immunomodulatory cytokines in serum and plasma of VS patients [38,72,73].

While our study focuses on cytokine concentrations in the CSF rather than serum or plasma, our results demonstrate a correlation between cytokine concentration in the CSF and the tumor volume, consistent with findings in the studies regarding serum. The concentrations of the cytokines CCL5, CCL18, and TGFB1 were higher in the CSF of the patients than in the CSF of healthy controls. For *TGFB1* and the receptors *TGFBR1* and *TGFBR2*, a higher expression was detectable in tumor tissue compared to vestibular nerve tissue, which is congruent with the CSF data. Moreover, varying concentrations were observed in patients with large and small tumors. The concentration of CCL2 and CCL18 was higher in the CSF of the patients with the large tumors than in the CSF of the patients with the small tumors, whereas it was the opposite for the cytokines TGFB1 and CCL5. Thus, the concentration of the cytokines CCL2, CCL5, CCL18, and TGFB1 in the CSF was associated with tumor size in patients. At present, no possibility to predict a tendency of growth in newly diagnosed VS is available.

However, the cohort of patients having CSF along with the growth rate was very small. Therefore, in order to be able to draw a conclusion about the interaction of the cytokines in CSF with growth rate, it would be necessary to determine the concentrations of cytokines in CSF from a larger number of patients having a growth rate. It has also not been conclusively clarified whether the controls are suitable as controls. Although controls were selected using exclusion criteria, CSF was taken from all controls due to neurological symptoms. These symptoms might be based on an undetected underlying disease that could influence the cytokine concentrations in the CSF. The different methods of collecting CSF in the control group compared to the VS patient group could also have an influence on the cytokine concentrations. It is not known whether anesthesia could influence the cytokine concentrations in the CSF. These factors could have an influence on the cytokines in the CSF of the VS patients and controls, which is why it cannot be conclusively answered whether the selected controls are suitable as such and can be compared with the VS patients.

Based on the data, it could be concluded that the immunomodulatory cytokines could have an influence on the tumor volume and the size progression of the VS. One possible mechanism, as described in more detail above, could be the interaction of TAMs with tumor cells via cytokines. However, no conclusions can be drawn from our data about possible mechanisms of VS development from the vestibular nerves. In contrast to the secretion and mRNA level data, conclusions about tumor progression in VS may not be possible solely based on CSF data. The cytokines in the CSF are not directly in contact with the VS tumor cells, as the tissue is separated from the surrounding tissue by a layer of connective tissue [74]. Therefore, the concentrations of cytokines in the CSF could provide new diagnostic possibilities for predicting the growth of VSs. To draw prognostic conclusions for new VS diagnoses and the growth prediction, further studies are needed to investigate the cytokine concentration in the CSF, not only at the time of surgery, but also at the time of diagnosis. In order to investigate whether the concentration of cytokines in CSF can also be used to diagnose VS, the CSF concentrations of cytokines in VS patients would have to be compared with the CSF concentrations of a larger group of controls. Patients with various diseases would have to be included in order to guarantee prognostic reliability for the future, as the actual patients are also suffering from underlying diseases. For both purposes, the collection of CSF from all VS patients and controls would have to be identical. Therefore, the CSF would need to be obtained using CSF puncture without anesthesia in all patients, as studies have revealed differences in protein concentration in lumbar and cerebral CSF [75,76].

Currently, there is no drug treatment option for sporadic VS [77]. Since the cytokines we investigated may have an impact on the tumor volume of VS, drug therapy and treatment options could be investigated. Indeed, several drugs have already been investigated for their efficacy in malignant tumors. There are approaches to bind cytokines, such as CCL2 and CCL5, and more likely their receptors by antibodies, such as Carlumab and BisCCL2/5i, and, thus, inhibit their effect [25,78,79,80,81]. Furthermore, the virustatic agent maraviroc is known to antagonize the CCL5 receptor and, thus, suppress the effect of CCL5 on it [28,82]. In trials, these drugs reduced the number of TAMs, tumor progression, and metastasis, and they improved overall survival in mice models. Further research could explore the possible influence of these substances on the growth behavior of primary VS cultures, aiming to identify potential therapeutic targets for sporadic VS.

## 5. Conclusions

Our results might suggest an influence of the immunomodulatory cytokines CCL5, CCL18, GDF15, and TGFB1 on tumor progression. Therefore, a mechanism similar to that in malignant tumors could be conceivable for the progression of benign VSs. The cytokines could attract macrophages into the tumor and polarize them to the M2 subtype. It is known from the literature that this type of TAMs is associated with tumor progression, which could explain the association between cytokines and tumor progression. Moreover, an immune escape mediated by CCL18 could not be disregarded. Moreover, cytokines secreted by tumor cells are secreted into the CSF of patients as well. Differences were found between control group and patients, but also between patients with large and small VSs. Due to the small number of patients, further investigations are needed to establish the correlation between growth rate and cytokine concentration in the CSF. In addition to new diagnostic possibilities resulting from the data, the cytokines may represent a starting point for new drug therapies.

## 6. Limitations

Due to the exploratory nature of the study, the number of patients in each group varied widely. Repeated MRI scans are required to calculate the growth rate. This follow-up can only be performed in patients with initially small tumors, which is why it was available from only a small group. The study was conducted on an exploratory basis, as no data for VSs are available on some of the investigated cytokines. Since the number of healthy subjects with CSF is limited, the control group for CSF comparison is small as well. A further limitation could be the different sampling method of the CSF, which could bias the true differences in the measured protein concentrations.

## Data Availability

The raw data and meta data of RNAseq analysis can be accessed in the Sequence Read Archive (SRA) of the National Center for Biotechnology (BioProject ID: PRJNA1150102). Further original contributions presented in the study are included in the article and Supplementary Materials; further inquiries can be directed to the corresponding author.

## Supplementary Materials

The following supporting information can be downloaded at: https://www.mdpi.com/article/10.3390/cancers16173002/s1, Table S1: Quantification of S100 and CD56 positive cells in VS primary cultures; Table S2: Antibodies used for immunofluorescence; Table S3: Homo sapiens specific primers used for quantitative Real-Time PCR; Table S4: Assessment of the correlations; Table S5: Baseline data for correlation analysis of investigated cytokines in 144 patients with sporadic VS; Table S6: Spearman’s r and *p*-values for correlation analysis of investigated cytokines and clinical parameters in 144 patients with sporadic VS; Table S7: Regression analysis of markers correlating significantly with tumor volume in correlation analysis of investigated cytokines in 144 patients with sporadic VS; Table S8: Baseline data for examining differential expression of studied markers in large and small VSs; Table S9: *p*-values for differential expression of studied markers in large and small VSs; Table S10: Medians and 75th percentiles of RNA transcription data of 10 tumors and corresponding nerves; Table S11: Baseline data for cytokine concentrations in CCS of 45 VS primary cultures and correlation analysis of primary cultures from 45 patients with sporadic VS; Table S12: CCS cytokine concentrations of investigated cytokines in 45 VS primary cultures; Table S13: Spearman’s r and *p*-values of investigated cytokines and clinical parameters in 45 VS primary cultures; Table S14: Regressions of investigated cytokines with tumor volume in 45 VS primary cultures; Table S15: Baseline data for correlation analysis of CSF samples from 52 patients with sporadic VS; Table S16: Baseline data of CSF control group; Table S17: Median concentrations of investigated cytokines in CSF of 52 patients and 14 healthy controls; Table S18: Correlations and *p*-values of investigated cytokines in CSF of 52 VS patients; Table S19: Regression of investigated cytokines with tumor volume in CSF of 52 VS patients.
