# Supplementary material for: Correlation of Immunomodulatory Cytokines with Tumor Volume and Cerebrospinal Fluid in Vestibular Schwannoma Patients
Source: Cancers (Basel). 2024 Aug 29;16(17):3002. doi: 10.3390/cancers16173002 (PMC11394145; doi:10.3390/cancers16173002)
Supplement: Supplementary file 1 [file cancers-16-03002-s001.zip › cancers-3145446-supplementary.pdf]

Supplementary Materials

Table S1. Quantification of S100^{+} and CD56^{+} cells in VS primary cultures.

Primary culture number	S100 [%]	CD56 [%]
1	82.20	76.24
2	94.95	88.08
3	92.22	86.42
4	90.07	79.20
5	89.63	94.47
6	86.69	71.28

Table S2. Antibodies used for immunofluorescence.

Antibody	Source / Isotype	Dilution	Manufacturer
S100	Mouse IgG	1:400	Cell signaling
CD56	Mouse IgG	1:400	Protein Simple
Anti-mouse-antibody	Goat Fab fragments	1:1000	Cell signaling

Table S3. Homo sapiens specific primers used for quantitative Real-Time PCR.

Gene symbol	Primer sequence 5′ to 3′ (Forward, Reverse)	Annealing temperature (°C)	Product length	Reference sequence
CD68	CGCAGCACAGTGGACATTCT, GGATCAGGCCGATGATGAGAG	60	236	NM_001251.3
CD163	AAAAAGCCACAACAGGTCGC, ATGGCCTCCTTTTCCATTCCA	60	322	NM_004244.5
MKI67	GATCGTCCCAGTGGAAGAGTT, ATTGCCTCCTGCTCATGGATT	60	276	NM_002417.5
CCL20	CGAATCAGAAGCAGCAAGCAAC, TTGCGCACACAGACAACTTTTT	61	161	NM_004591.3
CCL22	GCCGTGATTACGTCCGTTAC, GAGAATCATCTTCACCCAGGGC	61	155	NM_002990.5
IL10	AACCTGCCTAACATGCTTCGAG, GGTCTTGGTTCTCAGCTTGGG	61	202	NM_000572.3
IRF4	CCCAGCAGGTTCACAACTACA, TCGGCAGACCTTATGCTTGG	60	241	NM_002460.4
TGFB1	CAAGTGGACATCAACGGGTTC, GCAGAAGTTGGCATGGTAGC	60	270	NM_000660.7
GDF15	GGAGGTGCAAGTGACCATGT, ACCATGGGATTGTAGCTGGC	60	150	NM_004864.4
GAPDH	TCGTGGAAGGACTCATGACC, TTCCCGTTCAGCTCAGGGAT	60	172	NM_002046.7

Table S4. Assessment of the correlations.

Spearman's r	Assessment
0.000 – 0.199	Very weak correlation
0.200 – 0.399	Weak correlation
0.400 – 0.599	Moderate correlation
0.600 – 0.799	Strong correlation
0.800 – 1.000	Very strong correlation

Table S5. Baseline data for correlation analysis of investigated cytokines in 144 patients with sporadic VS.

**Variable**	**Total (n=144)**	**Percentage of all patients (n=175)**
Male	61	34.7
Female	83	47.2
Mean age (years) and standard deviation (SD)	51 ± 12.55 (18-77)	
Median age (years) and interquartile range (IQR)	52 (15.25)	
Koos grade		
I	4	2.3
II	47	26.7
III	51	29.0
IV	42	23.9
AAO-HNS[1] (hearing class)		
A	44	25
B	43	24.4
C	30	17.0
D	16	9.1
DS[2]	10	5.7
Without preoperative hearing class	1	0.06
Mean tumor volume (cm^3) and SD	4.0 ± 5.43 (0.1-37.0)	
Median tumor volume (cm^3) and IQR	2.3 (4.51)	
Mean growth rate (cm^3/year) and SD	0.7 ± 0.76 (0.04-2.7)	
Median growth rate (cm^3/year) and IQR	0.3 (0.83)	
Mean relative growth rate (%/year) and SD	2.9 ± 3.37(0.6-17.8)	
Median relative growth rate (%/year) and IQR	1.8 (1.71)	

[1] AAO-HNS: American Academy of Otolaryngology-Head and Neck Surgery

[2] DS: surditas for deafness

Table S6. Spearman's r and p-values for correlation analysis of investigated cytokines and clinical parameters in 144 patients with sporadic VS.

**Row**	**Column**	**Spearman's r**	**p-value**
Age at surgery	Koos	0.046	0.732
Age at surgery	Hearing class	0.386	**<0.001**
Koos	Hearing class	0.196	**0.049**
Age at surgery	Tumor volume	0.120	0.326
Koos	Tumor volume	0.891	**<0.001**
Hearing class	Tumor volume	0.220	**0.039**
Age at surgery	Relative growth rate	-0.125	0.679
Koos	Relative growth rate	0.124	0.679
Hearing class	Relative growth rate	-0.076	0.806
Tumor volume	Relative growth rate	0.224	0.403
Age at surgery	Growth rate	0.288	0.214
Koos	Growth rate	0.755	**<0.001**

Hearing class	Growth rate	0.234	0.350
Tumor volume	Growth rate	0.888	**<0.001**
Relative growth rate	Growth rate	0.364	0.098
Age at surgery	*CCL2*	-0.021	0.880
Koos	*CCL2*	-0.005	0.966
Hearing class	*CCL2*	-0.135	0.208
Tumor volume	*CCL2*	-0.115	0.350
Relative growth rate	*CCL2*	-0.192	0.467
Growth rate	*CCL2*	-0.331	0.147
Age at surgery	*CCL5*	0.087	0.467
Koos	*CCL5*	0.275	**0.003**
Hearing class	*CCL5*	0.078	0.526
Tumor volume	*CCL5*	0.267	**0.009**
CCL2	*CCL5*	0.369	**<0.001**
Relative growth rate	*CCL5*	-0.111	0.717
Growth rate	*CCL5*	0.049	0.880
Age at surgery	*CCL18*	0.020	0.884
Koos	*CCL18*	0.349	**<0.001**
Hearing class	*CCL18*	0.135	0.210
Tumor volume	*CCL18*	0.314	**0.002**
Relative growth rate	*CCL18*	0.120	0.696
Growth rate	*CCL18*	0.302	0.210
CCL2	*CCL18*	0.093	0.448
CCL5	*CCL18*	0.408	**<0.001**
Age at surgery	*CCL20*	-0.271	**0.004**
Koos	*CCL20*	-0.067	0.617
Hearing class	*CCL20*	-0.113	0.324
Tumor volume	*CCL20*	-0.049	0.732
Relative growth rate	*CCL20*	-0.029	0.933
Growth rate	*CCL20*	-0.251	0.324
CCL2	*CCL20*	0.412	**<0.001**
CCL5	*CCL20*	0.379	**<0.001**
CCL18	*CCL20*	0.067	0.617
Age at surgery	*CCL22*	-0.082	0.498
Koos	*CCL22*	0.194	0.054
Hearing class	*CCL22*	0.077	0.526
Tumor volume	*CCL22*	0.153	0.192
Relative growth rate	*CCL22*	0.188	0.493
Growth rate	*CCL22*	0.099	0.734
CCL2	*CCL22*	0.139	0.199
CCL5	*CCL22*	0.481	**<0.001**
CCL18	*CCL22*	0.216	**0.031**
CCL20	*CCL22*	0.427	**<0.001**
Age at surgery	*IL10*	-0.064	0.629
Koos	*IL10*	-0.045	0.732
Hearing class	*IL10*	0.022	0.880
Tumor volume	*IL10*	-0.108	0.399
Relative growth rate	*IL10*	0.010	0.966
Growth rate	*IL10*	-0.196	0.467

CCL2	*IL10*	0.579	**<0.001**
CCL5	*IL10*	0.380	**<0.001**
CCL18	*IL10*	0.146	0.179
CCL20	*IL10*	0.572	**<0.001**
CCL22	*IL10*	0.393	**<0.001**
Age at surgery	*TGFB1*	0.010	0.942
Koos	*TGFB1*	0.126	0.243
Hearing class	*TGFB1*	0.077	0.526
Tumor volume	*TGFB1*	0.148	0.199
Relative growth rate	*TGFB1*	-0.092	0.743
Growth rate	*TGFB1*	0.018	0.948
CCL2	*TGFB1*	0.174	0.085
CCL5	*TGFB1*	0.534	**<0.001**
CCL18	*TGFB1*	0.197	0.052
CCL20	*TGFB1*	0.357	**<0.001**
CCL22	*TGFB1*	0.588	**<0.001**
IL10	*TGFB1*	0.243	**0.011**
Age at surgery	*GDF15*	-0.047	0.734
Koos	*GDF15*	0.239	**0.023**
Hearing class	*GDF15*	-0.060	0.682
Tumor volume	*GDF15*	0.260	**0.020**
Relative growth rate	*GDF15*	-0.102	0.734
Growth rate	*GDF15*	0.005	0.980
CCL2	*GDF15*	0.324	**0.001**
CCL5	*GDF15*	0.192	0.079
CCL18	*GDF15*	-0.024	0.880
CCL20	*GDF15*	0.009	0.948
CCL22	*GDF15*	0.144	0.210
IL10	*GDF15*	0.089	0.493
TGFB1	*GDF15*	-0.008	0.951
Age at surgery	*IRF4*	-0.053	0.606
Koos	*IRF4*	0.285	**0.002**
Hearing class	*IRF4*	0.061	0.647
Tumor volume	*IRF4*	0.321	**0.001**
Relative growth rate	*IRF4*	-0.046	0.880
Growth rate	*IRF4*	-0.026	0.937
CCL2	*IRF4*	0.164	0.111
CCL5	*IRF4*	0.640	**<0.001**
CCL18	*IRF4*	0.278	**0.003**
CCL20	*IRF4*	0.351	**<0.001**
CCL22	*IRF4*	0.519	**<0.001**
IL10	*IRF4*	0.257	**0.007**
TGFB1	*IRF4*	0.540	**<0.001**
GDF15	*IRF4*	0.159	0.168
Age at surgery	*MKI67*	0.056	0.793
Koos	*MKI67*	-0.378	**<0.001**
Hearing class	*MKI67*	0.010	0.942
Tumor volume	*MKI67*	-0.313	**0.002**
Relative growth rate	*MKI67*	0.212	0.427

Growth rate	*MKI67*	-0.047	0.880
CCL2	*MKI67*	-0.244	**0.010**
CCL5	*MKI67*	-0.089	0.460
CCL18	*MKI67*	-0.087	0.475
CCL20	*MKI67*	-0.046	0.732
CCL22	*MKI67*	0.028	0.863
IL10	*MKI67*	-0.045	0.732
TGFB1	*MKI67*	0.222	**0.022**
GDF15	*MKI67*	-0.404	**<0.001**
IRF4	*MKI67*	0.026	0.877
Age at surgery	*CD163*	0.031	0.833
Koos	*CD163*	0.189	0.060
Hearing class	*CD163*	0.181	0.074
Tumor volume	*CD163*	0.200	0.068
Relative growth rate	*CD163*	0.039	0.901
Growth rate	*CD163*	0.142	0.629
CCL2	*CD163*	0.162	0.116
CCL5	*CD163*	0.348	**<0.001**
CCL18	*CD163*	0.208	**0.039**
CCL20	*CD163*	0.347	**<0.001**
CCL22	*CD163*	0.377	**<0.001**
IL10	*CD163*	0.281	**0.003**
TGFB1	*CD163*	0.453	**<0.001**
GDF15	*CD163*	0.016	0.918
IRF4	*CD163*	0.202	**0.042**
MKI67	*CD163*	0.181	0.073
Age at surgery	*CD68*	-0.048	0.729
Koos	*CD68*	0.368	**<0.001**
Hearing class	*CD68*	0.108	0.347
Tumor volume	*CD68*	0.344	**<0.001**
Relative growth rate	*CD68*	-0.051	0.880
Growth rate	*CD68*	0.312	0.191
CCL2	*CD68*	0.168	0.098
CCL5	*CD68*	0.462	**<0.001**
CCL18	*CD68*	0.398	**<0.001**
CCL20	*CD68*	0.246	**0.010**
CCL22	*CD68*	0.429	**<0.001**
IL10	*CD68*	0.235	**0.015**
TGFB1	*CD68*	0.502	**<0.001**
GDF15	*CD68*	0.100	0.442
IRF4	*CD68*	0.317	**<0.001**
MKI67	*CD68*	-0.052	0.696
CD163	*CD68*	0.666	**<0.001**

Significant p-values are written in bold (p≤0.05).

Table S7. Regression analysis of markers correlating significantly with tumor volume in correlation analysis of investigated cytokines in 144 patients with sporadic VS.

Coefficients	Estimate	Standard error	t-value	p-value
Growth rate	2.940	0.434	6.764	**<0.001**
CCL5	0.067	0.121	0.552	0.589

CCL18	-0.002	0.001	-2.049	0.058
GDF15	0.084	0.172	0.491	0.630
IRF4	1.063	0.358	2.966	**0.010**
MKI67	-0.228	0.392	-0.582	0.569
CD68	0.545	0.208	2.624	**0.019**

Multiple R-squared: 0.930; Adjusted R-squared: 0.898
F-statistic: 28.65 on 7 and 15 DF, p-value: **<0.001**

Table S8. Baseline data for examining differential expression of studied markers in large and small VS.

Variable	Small VS (n=20)	Percentage of all patients (n=175)	Large VS (n=20)	Percentage of all patients (n=175)	p-value
Male	8	4.5	10	5.7	0.75
Female	12	6.8	10	5.7	
Mean age (years) and SD	49 ± 9.87 (18-61)		49 ± 13.46 (25-75)		0.94
Median age (years) and IQR	51 (16)		49 (20.25)		
Koos grade					
I	3	1.7	0	0	
II	17	9.7	0	0	**<0.001**
III	0	0	0	0	
IV	0	0	20	11.4	
AAO-HNS[1] (hearing class)					
A	8	4.5	3	1.7	
B	8	4.5	5	2.8	**0.02**
C	2	1.1	5	2.8	
D	1	0.06	3	1.7	
DS[2]	1	0.06	4	2.3	
Mean tumor volume (cm^3) and SD	0.3 ± 0.16 (0.1-0.4)		13.8 ± 6.97 (7.5-37.0)		
Median tumor volume (cm^3) and IQR	0.3 (0.17)		12.5 (7.59)		**<0.001**

[1] AAO-HNS: American Academy of Otolaryngology-Head and Neck Surgery

[2] DS: surditas for deafness

Table S9. P-values for differential expression of studied markers in large and small VS.

	Median small tumors and IQR	Median large tumors and IQR	p-value
CCL2 mRNA level	1.54 (1.28)	0.98 (1.37)	0.056
CCL5 mRNA level	1.71 (1.17)	3.25 (4.40)	0.16
CCL18 mRNA level	0.69 (3.08)	5.26 (68.61)	**0.017**
CCL20 mRNA level	0.65 (0.52)	0.44 (0.47)	0.53
CCL22 mRNA level	2.01 (1.56)	2.50 (2.46)	0.25
IL10 mRNA level	1.41 (1.43)	0.51 (0.86)	0.13
IRF4 mRNA level	0.61 (0.74)	1.56 (1.44)	**0.013**
TGFB1 mRNA level	0.83 (0.54)	0.90 (0.79)	0.76
GDF15 mRNA level	0.55 (0.92)	1.19 (1.42)	0.1

Table S10. Medians and 75th percentiles of RNA transcription data of 10 tumors and corresponding nerves.

Gene	Tumor median [TPM]	Tumor 75th percentile [TPM]	Nerve median [TPM]	Nerve 75th percentile [TPM]	p-value
CCL2	30.33	48.74	451.84	619.04	**0.02**
CCL5	7.48	9.66	8.31	13.09	0.42
CCL18	0.00	0.06	0.00	0.00	0.12
CCL20	0.80	1.09	1.72	3.47	0.13
CCL22	0.23	0.41	0.22	0.37	0.60
IL10	0.48	1.07	1.32	2.80	**0.05**
GDF15	13.57	29.22	18.36	37.59	0.30
TGFB1	76.38	124.72	49.62	53.57	**0.006**
TGFB2	2.04	2.55	4.49	10.54	**0.04**
IRF4	0.20	0.31	0.46	0.8	0.15
MKI67	0.58	1.24	0.08	0.22	**0.02**
S100B	4355.96	4920.55	440.60	504.20	**0.007**
CCR2	1.09	1.60	0.29	0.85	**0.03**
CCR5	1.68	4.01	2.50	3.03	0.68
IL10RB	36.29	39.68	18.55	22.50	**<0.001**
TGFBR1	8.83	11.22	4.05	5.73	**0.002**
TFGBR2	75.37	162.89	31.82	44.15	**0.005**

Table S11. Baseline data for cytokine concentrations in CCS of 45 VS primary cultures and correlation analysis of primary cultures from 45 patients with sporadic VS.

Variable	Total (n=45)	Percentage of all patients (n=175)
Male	18	10.2
Female	27	15.3
Mean age (years) and SD	48 ± 12.69 (18-71)	
Median age (years) and IQR	48 (18)	
Koos grade		
I	0	0
II	16	9.1
III	13	7.4
IV	16	9.1
AAO-HNS[1] (hearing class)		
A	15	8.5
B	12	6.8
C	2	1.1
D	7	4.0
DS[2]	7	4.0
Without preoperative hearing class	2	1.1
Mean tumor volume (cm^3) and SD	3.6 ± 4.13 (0.1-18.0)	
Median tumor volume (cm^3) and IQR	2.47 (3.44)	

[1] AAO-HNS: American Academy of Otolaryngology-Head and Neck Surgery
[2] DS: surditas for deafness

Table S12. CCS cytokine concentrations of investigated cytokines in 45 VS primary cultures.

Cytokine	Median CCS concentration and IQR
CCL2	13199 (15899)
CCL5	12.90 (16.86)
CCL18	1.36 (3.99)
TGFB1	2092 (1705)
GDF15	378 (562)

Table S13. Spearman's r and p-values of investigated cytokines and clinical parameters in 45 VS primary cultures.

Row	Column	Spearman's r	p-value
Age at surgery	Hearing class	0.542	**0.002**
Age at surgery	Koos	-0.050	0.849
Hearing class	Koos	-0.016	0.993
Age at surgery	Tumor volume	0.097	0.803
Hearing class	Tumor volume	0.095	0.820
Koos	Tumor volume	0.912	**<0.001**
Age at surgery	*CCL2* qPCR	0.248	0.288
Hearing class	*CCL2* qPCR	0.012	0.993
Koos	*CCL2* qPCR	0.047	0.885
Tumor volume	*CCL2* qPCR	0.062	0.885
Age at surgery	*CCL5* qPCR	0.002	0.993
Hearing class	*CCL5* qPCR	-0.140	0.631
Koos	*CCL5* qPCR	0.065	0.885
Tumor volume	*CCL5* qPCR	0.001	0.993
CCL2 qPCR	*CCL5* qPCR	0.420	**0.025**
Age at surgery	*CCL18* qPCR	0.142	0.622
Hearing class	*CCL18* qPCR	0.195	0.470
Koos	*CCL18* qPCR	0.198	0.453
Tumor volume	*CCL18* qPCR	0.208	0.454
CCL2 qPCR	*CCL18* qPCR	0.668	**<0.001**
CCL5 qPCR	*CCL18* qPCR	0.410	**0.029**
Age at surgery	*TGFB1* qPCR	-0.047	0.885
Hearing class	*TGFB1* qPCR	-0.185	0.499
Koos	*TGFB1* qPCR	0.301	0.157
Tumor volume	*TGFB1* qPCR	0.217	0.436
CCL2 qPCR	*TGFB1* qPCR	0.100	0.769
CCL5 qPCR	*TGFB1* qPCR	0.349	0.079
CCL18 qPCR	*TGFB1* qPCR	-0.053	0.885
Age at surgery	*GDF15* qPCR	0.143	0.621
Hearing class	*GDF15* qPCR	-0.076	0.884
Koos	*GDF15* qPCR	0.455	**0.015**
Tumor volume	*GDF15* qPCR	0.436	**0.029**
CCL2 qPCR	*GDF15* qPCR	0.243	0.295
CCL5 qPCR	*GDF15* qPCR	0.150	0.600

CCL18 qPCR	*GDF15* qPCR	0.133	0.631
TGFB1 qPCR	*GDF15* qPCR	0.548	**0.002**
Age at surgery	CCL2 ELISA	0.147	0.600
Hearing class	CCL2 ELISA	-0.044	0.885
Koos	CCL2 ELISA	-0.049	0.885
Tumor volume	CCL2 ELISA	-0.048	0.885
CCL2 qPCR	CCL2 ELISA	0.626	**<0.001**
CCL5 qPCR	CCL2 ELISA	0.343	0.085
CCL18 qPCR	CCL2 ELISA	0.399	**0.033**
TGFB1 qPCR	CCL2 ELISA	0.154	0.600
GDF15 qPCR	CCL2 ELISA	0.055	0.885
Age at surgery	CCL5 ELISA	0.100	0.767
Hearing class	CCL5 ELISA	-0.214	0.402
Koos	CCL5 ELISA	0.065	0.885
Tumor volume	CCL5 ELISA	0.029	0.958
CCL2 qPCR	CCL5 ELISA	0.184	0.489
CCL5 qPCR	CCL5 ELISA	0.534	**0.002**
CCL18 qPCR	CCL5 ELISA	0.003	0.993
TGFB1 qPCR	CCL5 ELISA	0.403	**0.032**
GDF15 qPCR	CCL5 ELISA	0.372	0.053
CCL2 ELISA	CCL5 ELISA	0.411	**0.025**
Age at surgery	CCL18 ELISA	0.122	0.669
Hearing class	CCL18 ELISA	0.060	0.885
Koos	CCL18 ELISA	0.112	0.718
Tumor volume	CCL18 ELISA	0.058	0.885
CCL2 qPCR	CCL18 ELISA	0.487	**0.007**
CCL5 qPCR	CCL18 ELISA	0.247	0.288
CCL18 qPCR	CCL18 ELISA	0.681	**<0.001**
TGFB1 qPCR	CCL18 ELISA	0.039	0.906
GDF15 qPCR	CCL18 ELISA	0.059	0.885
CCL2 ELISA	CCL18 ELISA	0.663	**<0.001**
CCL5 ELISA	CCL18 ELISA	0.286	0.167
Age at surgery	TGFB1 ELISA	0.044	0.885
Hearing class	TGFB1 ELISA	0.001	0.993
Koos	TGFB1 ELISA	0.333	0.086
Tumor volume	TGFB1 ELISA	0.341	0.109
CCL2 qPCR	TGFB1 ELISA	-0.203	0.436
CCL5 qPCR	TGFB1 ELISA	0.002	0.993
CCL18 qPCR	TGFB1 ELISA	-0.265	0.244
TGFB1 qPCR	TGFB1 ELISA	0.547	**0.002**
GDF15 qPCR	TGFB1 ELISA	0.253	0.278
CCL2 ELISA	TGFB1 ELISA	0.214	0.387
CCL5 ELISA	TGFB1 ELISA	0.423	**0.022**
CCL18 ELISA	TGFB1 ELISA	0.133	0.631
Age at surgery	GDF15 ELISA	-0.010	0.993
Hearing class	GDF15 ELISA	-0.014	0.993
Koos	GDF15 ELISA	0.532	**0.002**
Tumor volume	GDF15 ELISA	0.473	**0.015**
CCL2 qPCR	GDF15 ELISA	-0.158	0.591

CCL5 qPCR	GDF15 ELISA	-0.133	0.631
CCL18 qPCR	GDF15 ELISA	-0.159	0.591
TGFB1 qPCR	GDF15 ELISA	0.373	0.053
GDF15 qPCR	GDF15 ELISA	0.431	**0.022**
CCL2 ELISA	GDF15 ELISA	0.170	0.520
CCL5 ELISA	GDF15 ELISA	0.287	0.167
CCL18 ELISA	GDF15 ELISA	0.108	0.730
TGFB1 ELISA	GDF15 ELISA	0.734	**<0.001**

Significant p-values are written in bold (p≤0.05).

Table S14. Regressions of investigated cytokines with tumor volume in 45 VS primary cultures.

**Coefficients**	**Estimate**	**Standard error**	**t-value**	**p-value**
GDF15 qPCR	1.637	0.282	5.806	**<0.001**
Multiple R-squared: 0.484, Adjusted R-squared: 0.4692<br>F-statistic: 33.71 on 1 and 36 DF, p value: **<0.001**				
GDF15 ELISA	2.909	2.808	1.036	0.307
Multiple R-squared: 0.028; Adjusted R-squared: 0.001919<br>F-statistic: 1.073 on 1 and 37 DF, p-value: 0.307				

Table S15. Baseline data for correlation analysis of CSF samples from 52 patients with sporadic VS.

**Variable**	**Patients (n=52)**	**Percentage of all patients (n=175)**	**Small VS (n=20)**	**Percentage of all patients (n=175)**	**Large VS (n=20)**	**Percentage of all patients (n=175)**	**p-value large vs. small VS**
Male	24	13.6	8	4.5	9	5.1	>0.999
Female	28	15.9	12	6.8	11	6.3	
Mean age (years) and IQR	49 ± 11.84 (18-71)		48 ± 7.8 (34-63)		53 ± 12.12 (30-71)		0.16
Median age (years) and SD	50 (14.25)		48 (12.25)		55 (16.5)		
Koos grade							
I	0	0	0	0	0	0	
II	13	7.4	11	6.3	0	0	**<0.001**
III	16	9.1	9	5.1	2	1.1	
IV	23	13.1	0	0	18	10.2	
AAO-HNS[1] (hearing class)							
A	17	9.7	6	3.4	4	2.3	
B	14	8.0	4	2.3	6	3.4	
C	3	1.7	1	0.06	2	1.1	0.93
D	9	5.1	5	2.8	3	1.7	
DS[2]	6	3.4	3	1.7	3	1.7	
Without preoperative hearing class	3	1.7	1	0.06	2	1.1	

Mean tumor volume (cm^3) and SD	4.3 ± 4.50 (0.1-20.1)	1.0 ± 0.65 (0.1-2.3)	8.2 ± 4.67 (3.1-20.1)	**<0.001**
Median tumor volume (cm^3) and IQR	2.8 (4.55)	0.8 (1.09)	7.3 (4.03)	
Mean growth rate (cm^3/year) and SD	0.9 ± 1.01 (0.03-3.7)	n. i.[3]	n. i.	
Median growth rate (cm^3/year) and IQR	0.6 (1.19)	n. i.	n. i.	
Mean relative growth rate (%/year) and SD	3.8 ± 4.58 (0.9-16.9)	n. i.	n. i.	
Median relative growth rate (%/year) and IQR	2.2 (2.35)	n. i.	n. i.	

[1] AAO-HNS: American Academy of Otolaryngology-Head and Neck Surgery;

[2] DS: surditas for deafness; [3] n.i.: not investigated due to the small sample number

Table S16. Baseline data of CSF control group.

Variable	Total (n=14)	Percentage of all control samples (n=14)
Male	5	35.7
Female	9	64.3
Mean age (years) and SD	54 (20-84)	
Median age (years) and IQR	50.5 (33.5)	

Table S17. Median concentrations of investigated cytokines in CSF of 52 patients and 14 healthy controls.

	Controls	Patients	Large VS	Small VS
Median CCL2 CSF concentration [pg/ml] and IQR	460.50 (177.50)	207.00 (126.00)	225.00 (176.25)	174.50 (73.75)
Median CCL5 CSF concentration [pg/ml] and IQR	1.17 (2.52)	91.45 (434.07)	7.99 (166.65)	334.50 (365.50)
Median CCL18 CSF concentration [pg/ml] and IQR	84.85 (67.47)	248.00 (263.75)	342.00 (309.50)	211.50 (156.00)

Median GDF15 CSF concentration [pg/ml] and IQR	227.00 (296.25)	181.00 (164.25)	230.00 (275.25)	169.00 (106.25)
Median TGFB1 CSF concentration [pg/ml] and IQR	173.00 (82.50)	342.50 (594.25)	233.50 (382.52)	620.00 (483.00)

Table S18. Correlations and p-values of investigated cytokines in CSF of 52 VS patients.

Row	Column	Spearman's r	p-value
Age at surgery	Hearing class	0.490	**0.002**
Age at surgery	Koos	0.111	0.715
Hearing class	Koos	0.025	0.935
Age at surgery	Tumor volume	0.227	0.430
Hearing class	Tumor volume	0.092	0.715
Koos	Tumor volume	0.880	**<0.001**
Age at surgery	Growth rate	0.802	**0.009**
Hearing class	Growth rate	0.263	0.715
Koos	Growth rate	0.887	**<0.001**
Tumor volume	Growth rate	0.958	**<0.001**
Age at surgery	Relative growth rate	0.294	0.715
Hearing class	Relative growth rate	-0.234	0.715
Koos	Relative growth rate	0.209	0.715
Tumor volume	Relative growth rate	0.343	0.656
Growth rate	Relative growth rate	0.531	0.319
Age at surgery	CCL2 CSF	-0.085	0.715
Hearing class	CCL2 CSF	-0.163	0.656
Koos	CCL2 CSF	-0.094	0.715
Tumor volume	CCL2 CSF	-0.093	0.715
Growth rate	CCL2 CSF	0.203	0.715
Relative growth rate	CCL2 CSF	0.154	0.758
Age at surgery	CCL5 CSF	-0.169	0.637
Hearing class	CCL5 CSF	-0.021	0.936
Koos	CCL5 CSF	-0.528	**<0.001**
Tumor volume	CCL5 CSF	-0.487	**0.002**
Growth rate	CCL5 CSF	-0.336	0.656
Relative growth rate	CCL5 CSF	-0.182	0.715
CCL2 CSF	CCL5 CSF	-0.101	0.715
Age at surgery	CCL18 CSF	0.172	0.637
Hearing class	CCL18 CSF	0.330	0.095
Koos	CCL18 CSF	0.042	0.899
Tumor volume	CCL18 CSF	0.159	0.657
Growth rate	CCL18 CSF	0.217	0.715
Relative growth rate	CCL18 CSF	0.441	0.522
CCL2 CSF	CCL18 CSF	0.106	0.715
CCL5 CSF	CCL18 CSF	-0.002	0.990
Age at surgery	TGFB1 CSF	-0.081	0.715
Hearing class	TGFB1 CSF	-0.002	0.990
Koos	TGFB1 CSF	-0.647	**<0.001**
Tumor volume	TGFB1 CSF	-0.645	**<0.001**

Growth rate	TGFB1 CSF	-0.322	0.677
Relative growth rate	TGFB1 CSF	-0.210	0.715
CCL2 CSF	TGFB1 CSF	-0.115	0.715
CCL5 CSF	TGFB1 CSF	0.881	**<0.001**
CCL18 CSF	TGFB1 CSF	0.028	0.926
Age at surgery	GDF15 CSF	0.346	0.061
Hearing class	GDF15 CSF	0.180	0.637
Koos	GDF15 CSF	0.038	0.902
Tumor volume	GDF15 CSF	-0.003	0.990
Growth rate	GDF15 CSF	-0.067	0.926
Relative growth rate	GDF15 CSF	-0.378	0.637
CCL2 CSF	GDF15 CSF	-0.068	0.758
CCL5 CSF	GDF15 CSF	-0.221	0.630
CCL18 CSF	GDF15 CSF	0.114	0.715
TGFB1 CSF	GDF15 CSF	-0.131	0.715

Significant p-values are written in bold (p≤0.05).

Table S19. Regression of investigated cytokines with tumor volume in CSF of 52 VS patients.

**Coefficients**	**Estimate**	**Standard error**	**t-value**	**p-value**
Growth rate	1.986	0.166	11.934	**<0.001**
CCL5 CSF	0.001	0.001	1.250	0.247
TGFB1 CSF	-0.015	0.014	-1.088	0.308
Multiple R-squared: 0.956; Adjusted R-squared: 0.9395<br>F-statistic: 57.9 on 3 and 8 DF, p-value: **<0.001**				
CCL5 CSF	0.003	0.004	0.839	0.406
TGFB1 CSF	-0.149	0.059	-2.505	**0.016**
Multiple R-squared: 0.244; Adjusted R-squared: 0.2111<br>F-statistic: 7.422 on 2 and 46 DF, p-value: **0.002**				
